# “Limits of Control” – Crucial Parameters for a Reliable Quantification of Viable *Campylobacter* by Real-Time PCR

**DOI:** 10.1371/journal.pone.0088108

**Published:** 2014-02-05

**Authors:** Nora-Johanna Krüger, Christiane Buhler, Azuka N. Iwobi, Ingrid Huber, Lüppo Ellerbroek, Bernd Appel, Kerstin Stingl

**Affiliations:** 1 National Reference Laboratory for *Campylobacter*, Federal Institute for Risk Assessment, Berlin, Germany; 2 Bavarian Health and Food Safety Authority, Oberschleißheim, Germany; Charité-University Medicine Berlin, Germany

## Abstract

The unsuitability of the “CFU” parameter and the usefulness of cultivation-independent quantification of *Campylobacter* on chicken products, reflecting the actual risk for infection, is increasingly becoming obvious. Recently, real-time PCR methods in combination with the use of DNA intercalators, which block DNA amplification from dead bacteria, have seen wide application. However, much confusion exists in the correct interpretation of such assays. *Campylobacter* is confronted by oxidative and cold stress outside the intestine. Hence, damage caused by oxidative stress probably represents the most frequent natural death of *Campylobacter* on food products. Treatment of *Campylobacter* with peroxide led to complete loss of CFU and to significant entry of any tested DNA intercalator, indicating disruption of membrane integrity. When we transiently altered the metabolic state of *Campylobacter* by abolishing the proton-motive force or by inhibiting active efflux, CFU was constant but enhanced entry of ethidium bromide (EtBr) was observed. Consistently, ethidium monoazide (EMA) also entered viable *Campylobacter*, in particular when nutrients for bacterial energization were lacking (in PBS) or when the cells were less metabolically active (in stationary phase). In contrast, propidium iodide (PI) and propidium monoazide (PMA) were excluded from viable bacterial cells, irrespective of their metabolic state. As expected for a diffusion-limited process, the extent of signal reduction from dead cells depended on the temperature, incubation time and concentration of the dyes during staining, prior to crosslinking. Consistently, free protein and/or DNA present in varying amounts in the heterogeneous matrix lowered the concentration of the DNA dyes at the bacterial membrane and led to considerable variation of the residual signal from dead cells. In conclusion, we propose an improved approach, taking into account principles of method variability and recommend the implementation of process sample controls for reliable quantification of intact and potentially infectious units (IPIU) of *Campylobacter* by real-time PCR.

## Introduction

Quantitative detection of *Campylobacter* is one of the most relevant issues for quality control of poultry products. For food legislative purposes, a viable *Campylobacter* forms a colony on an agar plate. Consistently, the ISO method 10272-2 detects colony forming units (CFU) per g or ml of sample material and is quite useful when applied to fresh samples (e.g. from the slaughterhouse), where *Campylobacter* just exited the intestine, the site of bacterial proliferation. However, the quantitative detection of the bacterial pathogen on poultry meat at retail is largely hampered by loss of in vitro culturability of *Campylobacter* due to cold and oxygen stress [1]. Whereas nearly 50% of all samples of German fresh chicken meat at retail were positive after enrichment (and probably recovery of transiently inactive *Campylobacter*), less than 5% of these samples were detected as *Campylobacter*-positive by direct plating (quantitative method) [2]. In contrast, it is not in doubt that these products are harmful to the consumer, since 20–30% of all cases of human Campylobacteriosis have been attributed to the direct consumption and/or handling of chicken products [3]. Hence, a culture-independent method for quantification of viable *Campylobacter* in food is indispensable for food control.

Ethidium monoazide (EMA) and propidium monoazide (PMA) are derivatives of ethidium bromide (EtBr) and propidium iodide (PI), respectively. They have been shown to intercalate into the DNA double helix and to covalently react with DNA upon light exposure [4]. Covalently bound EMA or PMA blocks PCR amplification. The use of EMA or PMA for real-time PCR-based discrimination of viable and dead bacterial cells is based on the assumption that these intercalator dyes do not cross the membrane of viable bacterial cells. Hence, only DNA within dead cells as well as free DNA are inactivated for PCR amplification [5].

A first study using EMA for detection of viable *Campylobacter* stated that EMA was passively excluded from the bacterial cells [6]. Recently, the use of PMA to inactivate DNA from dead *Campylobacter* cells was applied to rinses of fresh chicken carcasses from slaughterhouse [7]. Under the employed laboratory conditions, the method appeared to result in quite reproducible data and complete loss of PCR signal from dead *Campylobacter* detected on chicken carcasses. In a recent study, however, “rest signals” from dead *Campylobacter* after PMA treatment were observed [8]. Moreover, other studies reviewed in [9] indicated that PCR methods using intercalating dyes for the detection of several other viable microorganisms depend on a complex set of parameters.

We systematically monitored permeability properties of the common intercalating dyes across the bacterial membrane of *Campylobacter* (*C. jejuni* and *C. coli*) in different physiological states, while varying incubation temperatures from 0°C to 37°C. We showed that in contrast to PMA, EMA significantly entered viable cells, in particular those with low metabolic activity. Moreover, the process of live/dead discrimination using intercalating dyes largely depended on the composition of the matrices, which were relatively heterogeneous using chicken products as sampling material. Significantly, we observed that the real-time PCR signal from dead *Campylobacter* was dependent on temperature, time and matrix and was not completely erased by the intercalating dye, thus limiting accurate quantification of viable relative to dead *Campylobacter*. In conclusion, quantification of *Campylobacter* cells is limited unless carefully chosen sample process controls are established.

## Materials and Methods

### Strains and Cultivation Conditions

*Campylobacter* strains from −80°C stocks were cultured on Columbia blood agar (Oxoid) supplemented with 5% laked horse blood (Oxoid) for 18–48 h at 42°C under microaerobic atmosphere (5% O_2_, 10% CO_2_, rest N_2_). Strains were used as follows: *C. jejuni* DSM 4688, NCTC 11168, BfR-CA 9187 and *C. coli* DSM 4689, BfR-CA 9166, BfR-CA 9182. CFU were determined by serially diluting bacteria in buffered peptone water and plating on Columbia blood agar. Cell counts were microscopically determined using an improved Neubauer chamber (10 µm chamber depth, Marienfeld-Superior, Germany). Cation-supplemented Mueller-Hinton broth (CAMHB, Becton Dickinson, USA) was used for the fluorimetric assay.

### Fluorimeter Experiment

*Campylobacter* strains from a 24 h Columbia blood agar plate were inoculated at an OD_600_∼0.05–0.1 in Bolton liquid medium without selective supplements and shaken at 140 rpm and 42°C for 4–5 hours (exponential phase) or over night (stationary phase). Bacteria were harvested by centrifugation at 6000×g for 5 min and resuspended at OD_600_ 0.4 in CAMHB. Wells of a microtitre plate with optical bottom (Becton Dickenson, USA) were preloaded with 50 µl CAMHB supplemented with a 2-fold concentration of intercalating dye and effector, if indicated. Upon addition of 50 µl of the bacterial suspension, the microtitre plate was measured within 5 min in an infinite 200 PRO fluorimeter (Tecan Group Ltd., Switzerland) preheated to 37°C. Kinetics of fluorescence intensity was monitored at 37°C under occasional shaking using 530 nm and 600 nm as excitation and emission wavelength, respectively. Control wells without bacteria served as background fluorescence. The final concentration of EtBr and PI was 100 µM. As effectors, 100 µM of the protonophor carbonyl cyanide-m-chlorophenylhydrazone (CCCP), 150 µg/ml of phenyl-arginyl-β-naphthylamide (PAβN) or 5% H_2_O_2_ were added, if indicated.

### Preparation of Chicken Rinses and Sample Process Control (SPC)

Fresh chicken carcasses, chicken wings, chicken legs or frozen chicken carcasses were taken from retail and rinsed with PBS (135 mM NaCl, 2.7 mM KCl, 10 m Na_2_HPO_4_, 2 mM KH_2_PO_4_, pH 7.4) or 1% buffered peptone water (Merck, Germany) with or without 1% Tween-80. The volume of the rinse was adjusted to the weight of the chicken skin present on the sample: generally, ½ volume of rinse was applied to 1 v/w of chicken skin (e.g. 75 ml per whole chicken carcass, containing approximately 150 g of skin) for 1 min in a plastic bag. Alternatively, 1 v/w of chicken meat was treated with the corresponding volume of rinse for also 1 min in a plastic bag. This manipulation was rather undefined (light, medium, or strong interaction of the plastic bag with the surface of the chicken sample), leading to various heterogeneous matrices. In order to estimate the amount of organic matter in different chicken rinses, the protein concentration was measured after a freeze-thaw cycle using the Bradford method and bovine serum albumin as standard (Bio-Rad, USA).

For the preparation of SPC, *C. jejuni* grown for 24 h were resuspended in PBS at an OD_600_ of 0.2 (corresponding to ∼10^9^ bacterial counts/ml) and treated with 5% H_2_O_2_ for 1 h at room temperature. After inactivation, cells were centrifuged at 16.000×g for 5 min, resuspended in the same volume of PBS and stored on ice before use. 10 µl of dead *C. jejuni* (10^7^) were added to appropriate aliquots of the sample prior to the quantification procedure. In particular, two additional aliquots were prepared from the original sample, to which the SPC was added (10^7^ H_2_O_2_-treated dead *C. jejuni*). One of them was treated with PMA before crosslink (monitoring signal reduction from dead cells), the other one served as quantitative standard, accounting for putative DNA loss during sample isolation.

### EMA/PMA Treatment

EMA (Biotrend, Germany) and PMA (Biotium Inc., USA or GenIUL, Spain) were kept in stock solutions of 2–10 mM in 20% DMSO at −20°C. Samples of 1 ml were stained with the indicated concentration of the dyes. Chicken rinses were either stained directly or centrifuged for 3 min at 8000×g and resuspended in 1 ml PBS before addition of the dye. For the establishment of different temperature conditions, the cell suspensions were preincubated at the respective temperature for 10 min either in a water bath (25–37°C), in an incubator temperature block (10–20°C) or on ice (0°C). Subsequently, EMA or PMA was added and the incubation was further prolonged for 5 or 15 min in the dark prior to photoactivation for 15 min using the PHAST blue system at 100% light intensity (GenIUL, Spain). After photoactivation, the samples were centrifuged at maximal speed (16.000×g) for 5 min and stored at −20°C, if DNA extraction was not immediately performed.

### DNA Extraction

DNA was extracted with the GeneJET Genomic DNA Purification Kit (Thermo Fisher Scientific, USA) following the manufacturer’s instructions. In pre-tests it was confirmed by real-time PCR that DNA extraction was quantitative, using *Campylobacter* bacterial counts of 10^3^–10^7^ in PBS or in chicken rinse. DNA was eluted in 100 µl of elution buffer. If not otherwise stated, 10 µl of the purified DNA were used as template in the real-time PCR. Due to shearing and depurination of DNA, the quality of the DNA standard decreases with time, even when stored frozen [10]. Thus, the DNA standard (e.g. *C. jejuni* genomic DNA) was either prepared fresh or stably stored using the DNA protectant Qiasafe (Qiagen, Germany), dried and stored at room temperature in the dark. The concentration of the DNA was measured fluorimetrically, using the Quant-iT PicoGreen assay (Life Technologies, USA). Given the size of the *C. jejuni* chromosomal DNA of 1.6 Mb it was estimated that 1 ng of *C. jejuni* DNA contains 5.94×10^5^ chromosomal copies.

### Real-time PCR Detection

Real-time PCR was performed on an ABI Prism 7500 (Life Technologies, USA). Each 25 µl reaction mix contained 1 U Platinum Taq DNA Polymerase (Life Technologies, USA), 1× reaction buffer, 2.5 mM MgCl_2_, 0.4 mM of each dNTP (Thermo Fisher Scientific, USA) and the passive reference dye ROX (Life Technologies, USA). The 287 bp 16S rRNA target of *Campylobacter* spp. was detected according to [7], [11], using 500 nM forward (5′-CTGCTTAACACAAGTTGAGTAGG-3′) as well as reverse primer (5′-TTCCTTAGGTACCGTCAGAA-3′) (HPLC-grade, Sigma Aldrich, USA) and 100 nM of the dark-quenched hydrolysis probe (6FAM-TGTCATCCTCCACGCGGCGTTGCTGC-BBQ) (BBQ, BlackBerry quencher, TIB MOLBIOL, Germany). Inclusivity and exclusivity of the target detection was previously determined [11]. An appropriate DNA standard of *C. jejuni* chromosomal DNA, covering the complete range of measured chromosomal copy numbers (in 10-fold dilutions), was applied in duplicates in each real-time PCR assay and the efficiency was determined from the slope after linear regression. On an average, the efficiency of the real-time PCR was 89±4% and linearity ranged between 10^1^ to 10^6^ of chromosomal copies (three 16S rRNA gene targets are present per chromosomal copy), which is in agreement with the published data of the original method (efficiency 90% and linear range between 1.5×10^1^ to 10^7^) [12]. Note that linearity ranging at least up to 10^6^ chromosomal copies guarantees the quantitative detection of 1/10 of the extracted DNA of a sample spiked with 10^7^ *Campylobacter* cell counts.

As internal amplification control, 25 copies of the IPC-ntb2 detecting a 125-bp sequence of the *rbcMT-T* gene from *Nicotiana tabacum* with 300 nM forward (5′-ACCACAATGCCAGAGTGACAAC-3′) and reverse primer (5′-TACCTGGTCTCCAGCTTTCAGTT-3′) and 100 nM of the dark-quenched hydrolysis probe (Cy5-CACGCGCATGAAGTTAGGGGACCA-BBQ) was used [13]. After 3 min of Taq-Polymerase activation at 95°C, 45 PCR cycles of 15 s at 95°C, 1 min at 60°C and 30 s at 72°C were performed. Typical C_t_ values of 35–36 were obtained, unless PCR inhibition or depletion of PCR reagents was detected.

## Results

### Distinguishing Viable from Dead *Campylobacter* Cells

In order to show the unsuitability of the CFU parameter for quantitative assessment of the risk for infection by *Campylobacter* detected on food products, we systematically counted CFU, cell counts and DNA content from different physiological non-stressed states of *Campylobacter* cultures (Tab. 1). During exponential growth phase, roughly every forth cell multiplied to form a colony (cell counts/CFU  = 3.8, Tab. 1), while this was only observed for every tenth cell after incubation for 18–24 h (cell counts/CFU  = 12, Tab. 1). For cells after 40–48 h of incubation only one in ∼300 was able to form a CFU. Maximally 50% of the cells were stained with propidium iodide after 40–48 h of growth, indicating some loss of membrane integrity (cell death) but not explaining the magnitude of loss of CFU formation. In addition, *Campylobacter* cells tend to aggregate in stationary phase, leading to some apparent decrease of CFU. However, microscopic investigation showed that clustering of cells only marginally contributed to CFU reduction. Therefore, the data indicate that the number of non-growing, stationary and/or sub-lethally injured cells increased after prolonged incubation, even without artificial stress exposure. In contrast, the cell counts and the amount of DNA per OD∼0.2 were fairly stable (Tab. 1), resulting in an overall mean DNA content per cell of 0.8 chromosomal copies. Loss of DNA due to extraction from pure cultures was thereby minimal and ranged around 20%.

**Table 1:** Correlation of CFU, cell count and DNA content of *C. jejuni* cells.

Growth condition	Exponential phase cells	18–24 h Columbia blood agar	40–48 h Columbia blood agar
CFU/ml OD 0.2	2.5×10^8^±5.1×10^7^	9.2×10^7^±3.7×10^7^	4.0×10^6^±6.2×10^6^
cell counts/ml OD 0.2	9.5×10^8^±2.0×10^8^	1.1×10^9^±3.5×10^8^	1.3×10^9^±5.0×10^8^
DNA/ml OD 0.2	1.46 µg ±0.5	1.54 µg ±0.34	1.56 µg ±0.49
chrom. copies/ml OD 0.2	8.6×10^8^±2.1×10^7^	9.0×10^8^±1.8×10^8^	9.3×10^8^±2.9×10^8^
chrom. copies/cell count	0.9	0.8	0.7
cell counts/CFU	3.8	12.0	325

Exponential phase cells were grown microaerobically in Bolton broth without selective supplements for 4–5 h at 140 rpm (inoculation from an 18–24 h Columbia agar plate). DNA amount and calculated chromosomal copies correspond to DNA extracted by a commercial silica column kit. Data stem from at least five independent experiments.

Our data confirm that the CFU does not represent a reliable parameter for reproducible quantification of *Campylobacter*. This is, in principle, true for all bacteria but in particular for those with fastidious growth requirements. Hence, we suggest that the quantification of “intact and putatively infectious units” (IPIU) of *Campylobacter* would be more suitable to reflect the potential risk for infection by food products. These IPIU are defined as harbouring an intact membrane, which is non-permeable by the widely accepted live/dead discriminatory DNA dye, propidium iodide. They comprise but are not limited to the “viable but non-culturable bacteria” (VBNC).

A microaerobic bacterium is confronted by oxidative and cold stress outside of the intestine. Therefore, damage caused by oxidative stress probably represents the most frequent natural death of *Campylobacter* on food products, meaning “physiological death”. *Campylobacter* cells were heat inactivated for 5 min at 95°C or 10 min at 70°C or killed by oxidative stress with 5% H_2_O_2_ for 1 hour at room temperature. No residual CFU was detected (>log 7 inactivation), irrespective of the inactivation treatment. From these inactivated bacteria, *Campylobacter* DNA was detected by real-time PCR using the 16S rRNA target [7]. Upon oxidative stress or heat inactivation at 70°C for 10 min, the C_t_ value of the total DNA extracted from killed bacteria remained fairly constant compared to untreated samples. Treatment at 95°C for 5 min, however, resulted in significant increase in C_t_ values (corresponding to Δlog copies of 0.68±0.53, Tab. 2), indicating that DNA was significantly liberated from the cells.

**Table 2:** Loss of DNA upon cell inactivation.

Treatment of 10^6^ cells in PBS	Δlog copies
None	0
Oxidative stress (5% H_2_O_2_, 1 h)	0.18±0.14
Heat inactivation (70°C, 10 min)	0.31±0.33
Heat inactivation (95°C, 5 min)	0.68±0.53

Data stem from at least seven independent experiments using bacterial suspensions from all growth phases.

### EtBr does not Adequately Discriminate between Viable and Membrane-compromised *Campylobacter* Cells, whereas PI does

**Figure 1:**
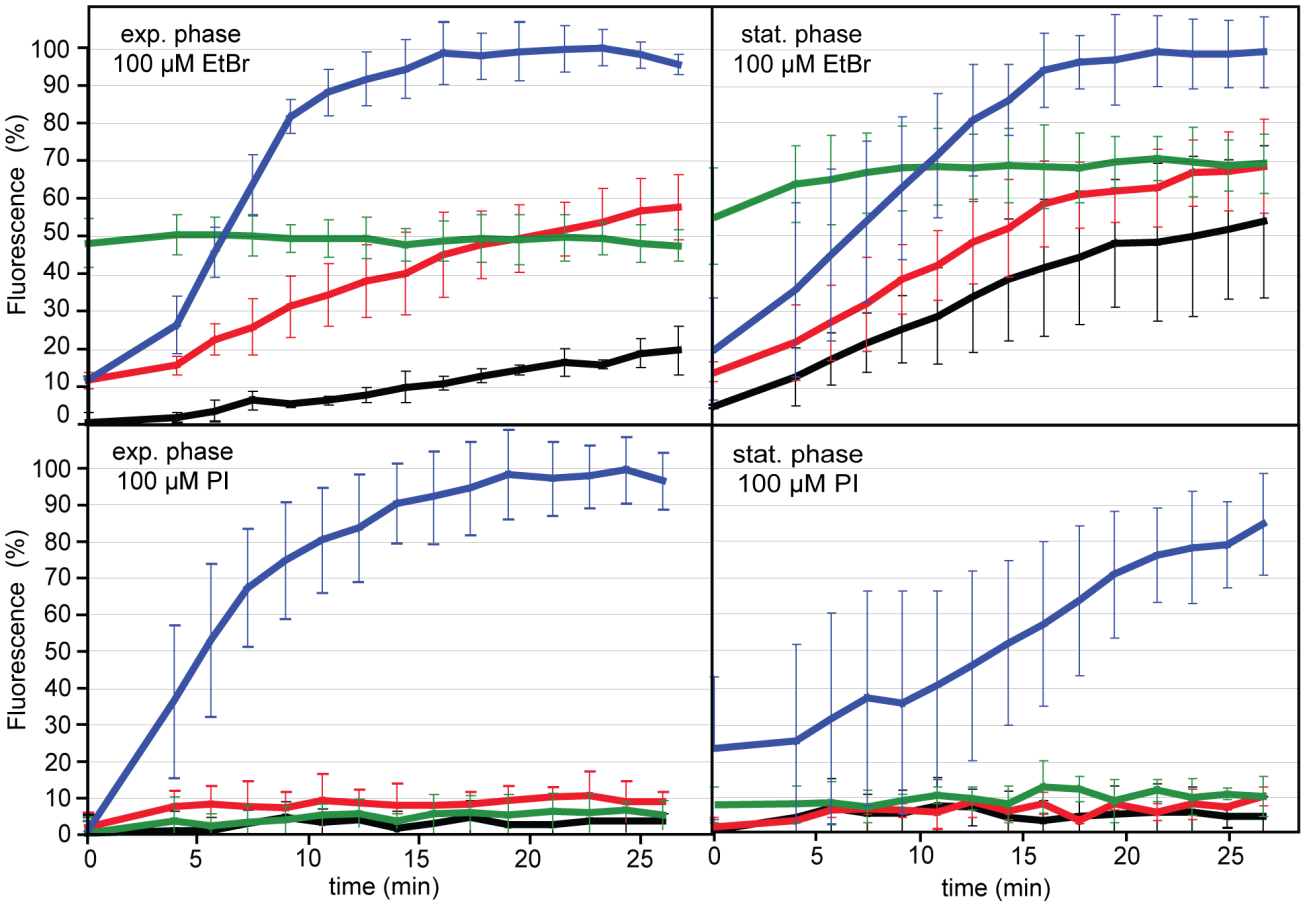
Fluorimetric analysis of EtBr and PI entry into *C. jejuni* DSM 4688 at different metabolic states and during oxidative stress. Cells were grown to either exponential phase (OD 0.3–0.7) or stationary phase (OD 1.5–1.7) and diluted to OD 0.2 for the fluorimetric assay. At the timepoint  = 0, *C. jejuni* were confronted with either 100 µM EtBr (upper panel) or 100 µM PI (lower panel) and with distinct effectors. Black, control without effector; red, 150 µg/ml of the efflux inhibitor PAβN; green, 100 µM of the protonophor CCCP; blue, 5% H_2_O_2_, leading to *C. jejuni* cell death. While EtBr enters *C. jejuni* depending on the metabolic state, PI is passively excluded from viable cells. Mean fluorescence intensities (in 100% of the maximal fluorescence of the respective dead (H_2_O_2_) cell suspension reached after saturation (for stationary phase in the presence of PI 100% value was reached after 40 min)) and standard deviations are depicted from at least three independent experiments.

For the evaluation of the discrimination power between viable and dead cells, using the two common intercalating reagents, EMA and PMA, we performed membrane permeability tests. The principle of the assay is the measurement of fluorescence development upon entry of the intercalating dye into the cytoplasmically located nucleic acids. Since the monoazide groups of EMA and PMA decompose to highly reactive nitrene groups upon light induction, precluding real-time fluorescence detection, we chose their analogues, EtBr and PI for this experimental approach (Fig. 1).

At the start of the experiment, dye and effector were added to the cell suspension and the fluorescence development was measured at an excitation wavelength of 530 nm and an emission wavelength of 600 nm. In order to obtain viable but metabolically inactive cells, we added the protonophor CCCP. This effector abolishes the proton motive force, and, consequently, leads to loss of ATP in *Campylobacter* [14]. In addition, we tested the efflux inhibitor, PAβN, which was shown to inhibit active export of antibiotic drugs from *C. jejuni* cells [15]. As mentioned above, treatment of the bacteria with 5% H_2_O_2_ for 1 hour led to a >log 7 decrease in CFU. In contrast, efflux inhibited or proton-motive force deprived cells generated similar CFU as the control (2.5×10^8^ CFU/ml ±5.1×10^7^) after 1 hour of exposure to either 150 µg/ml PAβN (3.8×10^8^ CFU/ml ±2.9×10^8^ CFU/ml) or 100 µM CCCP (4.5×10^8^ CFU/ml ±2.6×10^8^).

Shown by the increase in fluorescence with time, EtBr even entered into the control cells, although the kinetics for exponential cells was slow compared to the significantly higher uptake of the dye in stationary phase cells (Fig. 1, upper panel, black lines). Hence, exponential cells with higher metabolic activity appeared to exhibit higher efflux activity for EtBr. Consistently, the addition of the efflux inhibitor PAβN led to a drastic increase in EtBr influx (Fig. 1, upper panel, red lines), in particular in the exponential phase cells. Likewise, metabolically inactive cells incubated with CCCP showed immediate high influx of EtBr (Fig. 1, upper panel, green lines). As control, the H_2_O_2_ stressed cells showed the highest influx of EtBr. The high variation of fluorescence data in stationary phase cells hint at the heterogeneous state of bacteria in this phase of growth.

Most important and unlike EtBr, PI did not enter viable cells, irrespective of the growth phase and/or the metabolic state of the cells (Fig. 1, lower panel). The slightly different kinetics of the dyes’ influx in H_2_O_2_-treated exponential and stationary phase cells might suggest a higher oxidative stress resistance of *Campylobacter* upon entry into stationary phase. From the data, we conclude that after 1 h of incubation with H_2_O_2_, a “plateau” of PI fluorescence is reached for most *Campylobacter* cultures. Thus, this treatment was applied for the production of “physiologically dead” cells in further experiments, in particular since we observed variable DNA loss from cells upon heat inactivation (see above).

For extending the interpretation of these data to *Campylobacter* strains other than *C. jejuni* DSM 4688, we repeated the fluorimetric analysis with *C. jejuni* NCTC 11168 and *C. jejuni* BfR-CA 9187 and with *C. coli* DSM 4689, *C. coli* BfR-CA 9166 and *C. coli* BfR-CA 9182. The results were similar. Influx of PI into viable bacterial cells was never observed, while entry of EtBr was variable, according to the metabolic state of the bacteria (data not shown).

### Membrane Permeability of EMA and PMA Resembles that of their Analogues

In order to check whether EMA and PMA exhibited similar membrane permeability properties in *Campylobacter* compared to their analogues, *C. jejuni* DSM 4688 cells in exponential phase were incubated for 1 hour in CAMHB supplemented either with 100 µM CCCP or 150 µg/ml PAβN under microaerobic atmosphere at 37°C or with 5% H_2_O_2_ in PBS. Subsequently, the cells were harvested by centrifugation, resuspended in PBS and incubated with either 20 µM EMA or PMA for 15 min at room temperature, in the dark before light-induced crosslink. After DNA extraction, the ΔC_t_ value in the presence and absence of intercalating dye was measured by real-time PCR.

Analogous to PI, the data showed that PMA detected viable *Campylobacter* independent of their current physiological state (ΔC_t_  = 0.4±0.6, control cells; ΔC_t_  = 0.6±0.4, CCCP-treated cells; ΔC_t_  = 0.9±0.5, PAβN-treated cells, Tab. 3) and blocked DNA for real-time PCR detection in dead cells (ΔC_t_  = 8.7±3.1 (n = 10), H_2_O_2_-treated cells). In contrast, EMA already significantly blocked DNA for real-time PCR detection in the control cells (ΔC_t_  = 8.8±2.4, Tab. 3). The latter effect was surprising concerning the magnitude of PCR signal loss for viable cells but was reproducible, irrespective of the growth state of the cells. However, when EMA staining was performed for only 5 min instead of 15 min and in peptone water instead of PBS, loss of signal in viable cells could be reduced to ΔC_t_  = 1.2±0.2 (ex. phase cells and 18–24 h cells) and to ΔC_t_  = 4.4±2.8 (stationary phase cells after incubation for 40–48 hours), respectively. Similar results were obtained with chicken rinse, where EMA intercalation into DNA of viable cells was dependent on the metabolic state (ΔC_t_  = 1.1±0.8, ex. phase and 18–24 h cells versus 4.0±0.6 for 40–48 h cells, Tab. 3). In conclusion, permeability of EMA in viable *Campylobacter* cells was highly variable and depended on the medium of incubation (PBS, peptone water or chicken rinse), the incubation time and the state of the cells, probably reflecting the magnitude of active bacterial efflux of the dye (Tab. 3). In contrast, PMA was efficiently excluded from viable *Campylobacter*, which harbour an intact membrane, irrespective of active efflux activity of the bacteria.

**Table 3:** Loss of real-time PCR signal (ΔC_t_ value) from viable bacteria by staining with EMA or PMA.

Stainingconditions	bacteria	20 µM EMA (5 min)	20 µM EMA (15 min)	20 µM PMA (15 min)
PBS	all	6.1±3.7 (n = 5)	8.8±2.4 (n = 9)	0.4±0.6 (n = 13)
	all, CCCP-treated	n. d.	n. d.	0.6±0.4 (n = 5)
	all, PAβN-treated	n. d.	n. d.	0.9±0.5 (n = 5)
BPW	all			0.2±0.3 (n = 6)
	ex.phase +24 h cells	1.2±0.2 (n = 5)	1.9±0.4 (n = 8)	
	48 h stat. Cells	4.4±2.8 (n = 4)	6.0±2.5 (n = 5)	
cRinse	all			0.3±0.3 (n = 7)
	ex.phase +24 h cells	n. d.	1.1±0.8 (n = 12)	
	48 h stat. cells	n. d.	4.0±0.6 (n = 5)	

10^6^ viable *C. jejuni* cells were spiked into 1 ml of either phosphate buffered saline (PBS), buffered peptone water (BPW) or chicken rinse (cRinse). Staining was performed for 5 or 15 min in the dark at room temperature. Mean ΔC_t_ values (with and without dye) ± standard deviation are shown.

ex.phase +24 h cells, bacteria grown for 18–24 h on Columbia blood agar or exponentially in liquid medium; 48 h stat. cells, bacteria grown for 40–48 h on Columbia blood agar; all, viable bacteria without differentiation of the growth phase; n. d., not determined; n, number of tested samples.

### Dependence of the Incubation Temperature and Time in the Presence of the Dye before Crosslink

The magnitude of DNA inactivation for PCR detection in bacterial cells largely depends on the kinetics of membrane permeability for the respective dye. Without regard to active export conducted by viable cells, this kinetics is mainly governed by diffusion. Therefore, we questioned the influence of temperature and time on signal reduction by 20 µM EMA or PMA in viable and oxidative stressed dead cells (H_2_O_2_-treated). We used either exponential or 18–24 h cultures of *C. jejuni* DSM 4688, which were resuspended in PBS and pre-incubated for 10 min at the respective temperature before staining with either EMA or PMA for 5 or 15 min.

**Figure 2:**
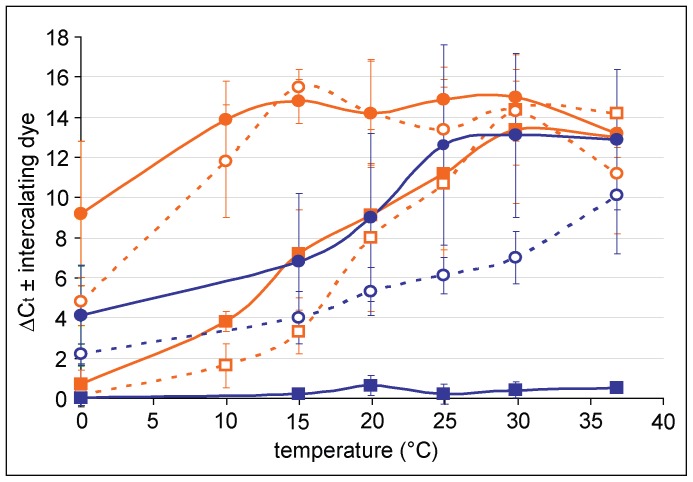
Time and temperature dependence of dye entry into viable and dead *Campylobacter* cells stained in PBS at 10^6^ bacterial counts/ml. ΔC_t_-values from real-time PCR of DNA isolated from bacteria in the presence and absence of 20 µM of the respective intercalating dye. Squares, viable bacteria; circles, dead bacteria; blue, PMA; orange, EMA; open symbols with dotted lines, 5 min of incubation; closed symbols with filled lines, 15 min of incubation. Mean values and standard deviations are depicted from at least three independent experiments.

As shown above, PMA was efficiently excluded from viable *Campylobacter*, while significant entry of EMA was observed (Fig. 2). As expected for a mainly diffusion-limited process, entry of EMA into viable cells was more pronounced with prolongation of incubation time and with increasing temperatures. On ice, EMA was efficiently excluded from viable cells. However, under these conditions DNA from dead cells was less efficiently blocked by EMA (ΔC_t_  = 4.8±1.2 (5 min) and ΔC_t_  = 9.2±3.6 (15 min), Fig. 2). Using chicken rinse with high organic matter, signal reduction from dead cells upon staining with EMA for 15 min was even less (ΔC_t_  = 2.7±0.9 (n = 4)) and insufficient for proper live/dead discrimination. As expected, entry of PMA into dead cells was also time- and temperature-dependent. Optimal conditions were 15 min at 30°C of incubation with reduction of dead cell signal at ΔC_t_  = 13.1±4.1 (Fig. 2) observed.

### Decrease of the Actual Dye Concentration at the Bacterial Membrane by Matrix Effects Lead to Variability of Dead Cell Signal Reduction

Given the fact that membrane permeability is governed by diffusion, the actual concentration of the dye at the membrane is a crucial parameter. Above, we showed that the use of peptone water instead of PBS significantly changed the behaviour of EMA entry into viable cells, probably because *Campylobacter* were energized and actively exported EMA. In addition, it is expected that the actual concentration of dyes at the bacterial membrane is strongly altered by the composition of the matrix. In particular, tissue and released DNA can absorb intercalating dyes, reducing the real concentration of dyes at the bacterial membrane. From our experience, the preparation of chicken rinse results in the production of very heterogeneous matrices. As starting material, whole fresh chicken carcasses, chicken legs, chicken wings, chicken meat as well as frozen chicken carcasses were used. PBS was added at approximately ½ v/w of skin material. In the case of chicken meat a 1 v/w ratio of PBS versus meat was used. Moreover, the kinetics of “rinsing” varied from person to person, as the “contact force of the plastic bag with the sample surface” is not standardized. We estimated variations of the load of organic material in prepared chicken rinses by determination of the free protein concentration. The measured protein concentrations of prepared chicken rinses ranged widely from 0.3 mg/ml to over 30 mg/ml, thus reflecting a potential 100-fold variation in protein load.

In contrast to EMA, using PMA guaranteed that viable cells were not inactivated for real-time PCR detection, irrespective of the matrix and the metabolic state of the viable bacteria (see above). Given that the intercalating dye stays out of viable cells, it is of major importance to rely on a sufficient signal reduction of dead cells. The efficiency of signal reduction was affected by the presence of organic matter in rinse matrices. When 20 µM PMA was used for 15 min at 20°C, only a mean reduction of ΔC_t_  = 3.8±1.6 was observed for dead cells in chicken rinses (Tab. 4). Increasing the temperature to 30°C led to an enhancement of dead cell signal reduction to ΔC_t_  = 6.5±1.9. Centrifuging and resuspending the sample in PBS prior to staining even improved dead cell signal reduction to ΔC_t_  = 7.7±2.0 at 20°C and to ΔC_t_  = 8.9±2.1 at 30°C. Using higher concentrations of PMA (50 and 100 µM) gave results in accordance with the observations for 20 µM PMA, but with generally increased dead cell reduction (up to ΔC_t_  = 11.2±1.7 for 100 µM PMA at 30°C (Tab. 4)). It is notable that the degree of dead cell reduction was not dependent on the total amount of dead cells spiked in chicken rinse in the range of 10^4^–10^7^ bacteria (data not shown). This result is understandable, since these amounts of dead bacteria are relatively low compared to the amount of organic matter from chicken tissue, putatively representing the largest “sink” for dye absorbance during incubation under these conditions. Table 4 depicts data from our set of experiments, meant to demonstrate principles of matrix-dye interaction. It does not represent absolute values for practical usage. This is why the implementation of a proper “samples process control” (SPC) for each quantification setup is recommended. This SPC serves for the monitoring of variable process parameters, including matrix effects, dye concentration, crosslink conditions and DNA extraction efficiency (see below).

**Table 4:** Reduction of dead cell signal of *C. jejuni* in chicken rinses by PMA.

PMA (µM)	centrifugation	ΔC_t_ ± PMA (20°C)	ΔC_t_ ± PMA (30°C)
20	−	3.8±1.6 (n = 17)	6.5±1.9 (n = 13)
	+	7.7±2.0 (n = 4)	8.9±2.1 (n = 7)
50	−	5.1±2.5 (n = 8)	9.9±1.3 (n = 10)
	+	n. d.	10.8±1.2 (n = 12)
100	−	9.1±1.2 (n = 5)	10.6±1.7 (n = 13)
	+	n. d.	11.2±1.7 (n = 13)

10^6^–10^7^ dead *C. jejuni* cells (H_2_O_2_-treated) were spiked into 1 ml of chicken rinse. Staining was performed either by direct addition of PMA or after centrifugation and resuspension in 1 ml PBS (+ centrifugation). All samples were incubated for 15 min in the dark at the respective temperature before photoactivation. Mean ΔC_t_ values (with and without PMA) ± standard deviation are shown; n. d., not determined; n, number of tested samples.

### Quantification of Viable *Campylobacter* in the Presence of Excess Dead Bacteria

**Figure 3:**
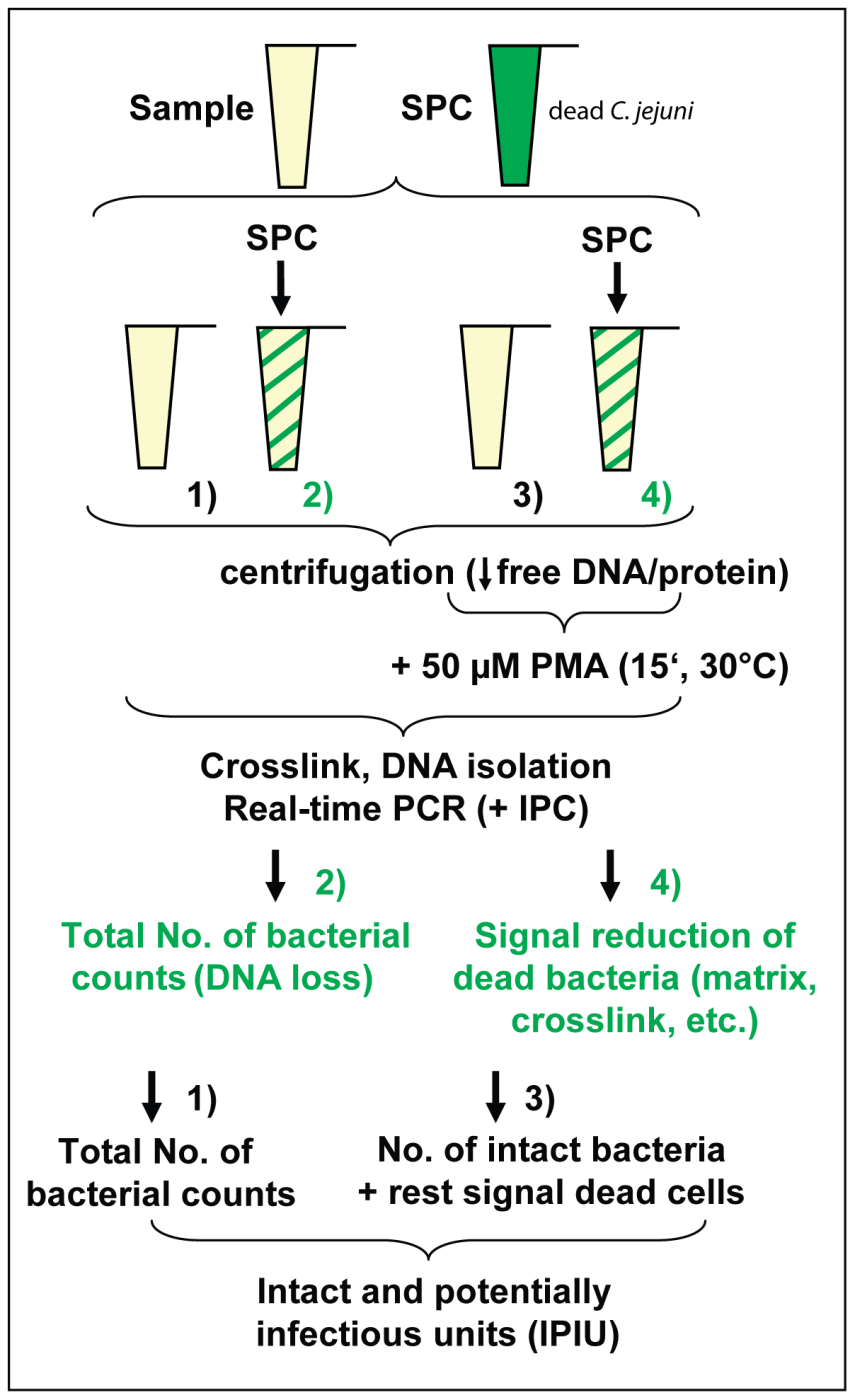
Schematic view of the quantification approach. The sample is aliquoted to four tubes, two of which (tube 2 and 4) are spiked with the sample process control (SPC), a distinct number of dead *C. jejuni* (e. g. 10^7^ H_2_O_2_-treated *C. jejuni*). In order to lower the amount of free DNA and/or protein, potentially interfering with the dose-effect relation of PMA, the samples are centrifuged and resuspended into PBS. After PMA addition, diffusion of PMA into dead bacteria and intercalation into DNA is optimal at 30°C for 15 min in the dark prior to crosslink. After DNA extraction, the target is quantified by real-time PCR, including an IPC for amplification control. SPC in tube 2 serves as calibration standard for 10^7^ bacterial counts, relative to which C_t_ values can be translated to bacterial counts using the slope of the DNA standard curve. The ΔC_t_ of tube 2 versus tube 4 tests for sufficient signal reduction from dead cells (including matrix effects and crosslink conditions). If signal reduction from dead cells significantly exceeds the reduction of the signal of the sample in the presence of PMA, the number of “intact and potentially infectious units” (IPIU) can directly be calculated from the C_t_ value of tube 3, which is translated to bacterial counts as mentioned above.

DNA loss during extraction was monitored by implementation of a SPC during the whole procedure. As shown above, we observed that the OD_600_ correlated well with bacterial counts and DNA amount, even if *Campylobacter* cells were grown to different growth phases (Tab. 1). Thus, we used a suspension of dead *C. jejuni* (e.g. H_2_O_2_-treated) of a defined cell number, significantly exceeding the total number of “expected” native *Campylobacter* in the sample (e.g. 10^7^ bacterial counts). This aliquot (Fig. 3, tube 2) was used for calibration, relative to which the unknown amount of native *Campylobacter* counts were calculated on the basis of the slope of the C_t_ values obtained from the DNA standard.

For the confirmation that the matrix-dependent residual signal from dead cells was negligible, we implemented a second SPC spiked with dead *C. jejuni* (e.g. 10^7^), this time in the presence of PMA (Fig. 3, tube 4). The variability in reduction of dead cell signal is negligible as long as the ΔC_t_ of dead cell reduction (from the SPC) significantly exceeds the measured ΔC_t_ reduction of the sample in the presence of PMA. Assuming a realistic real-time PCR efficiency for the 287 bp 16S rRNA target [7] of 90% (i. e. ΔC_t_  = 3.6 which corresponds to a log 1 target change) and a signal reduction of dead cells around ΔC_t_  = 10; the quantification of viable *Campylobacter* would be feasible in excess of maximally log 2.5 dead cells, (i. e. 0.3% of viable cells could be accurately quantified in the context of 99.7% of dead bacteria (see also Tab. 5)).

**Table 5:** Proof of principle: Quantification of viable *Campylobacter* in the context of excess dead *Campylobacter* in chicken rinses.

			cRinse_low_		cRinse_high_	
live/dead	PMA (µM)	centrifugation	log IPIU	Δlog deviation	log IPIU	Δlog deviation
log 3/log 5.5	*20*	*−*	*3.74*	*0.74*	*4.71*	*1.71*
	50	+	2.83	−0.17	3.38	0.38
	100	+	2.86	−0.14	3.15	0.15
log 3/log 5	*20*	*−*	*3.56*	*0.56*	*4.16*	*1.16*
	50	+	2.8	−0.2	3.15	0.15
	100	+	2.92	−0.08	3.15	0.15
log 2/log 4	*20*	*−*	*2.46*	*0.46*	*3.09*	*1.09*
	50	+	1.93	−0.07	2.28	0.28
	100	+	2.26	0.26	2.27	0.27

Using the method depicted in Fig. 3, we quantified log 2–3 viable *C. jejuni* in the context of log 2–2.5 excess of dead *C. jejuni* in two different chicken rinses (cRinse) with low (∼1.4 mg/ml of protein) and high concentrations of organic matter (∼7.8 mg/ml of protein). In *italics*, PMA was directly added to the samples and incubated for 15 min at 20°C; all other samples were centrifuged and resuspended in PBS prior to PMA staining for 15 min at 30°C. ΔC_t_ of signal reduction from dead bacteria (SPC, Fig. 3, tubes 2 and 4) in the presence of 50–100 µM PMA was >10 for both matrices, whereas 20 µM PMA (without prior centrifugation before staining) resulted in ΔC_t_  = 4.2 (cRinse_low_) and ΔC_t_  = 2.1 (cRinse_high_). IPIU, intact and potentially infectious units.

The internal amplification control (IPC) was routinely integrated in the real-time PCR assay, in order to detect PCR inhibitors, for example high amounts of non-target DNA co-isolated from the matrix. This is of particular importance for detection of PCR inhibition in those samples in which PMA was applied and where high expected C_t_ values might mask PCR inhibition.

For a proof of principle, we tested if viable *Campylobacter* could be detected in excess of log 2 or log 2.5 dead bacteria using 20 µM PMA at 20°C without prior sample centrifugation or, alternatively, using 50 µM or 100 µM at 30°C with prior sample centrifugation (in order to lower the amount of organic matter in the sample) (Tab. 5). As SPC, dead *C. jejuni* (H_2_O_2_-treated for 1 h in PBS at OD  = 0.2, corresponding to 10^9^ bacterial counts/ml) were spiked at 10^6^ (or 10^7^) cell counts in two matrix aliquots, one of them treated with PMA before crosslink, the other serving as quantitative standard for 10^6^ (or 10^7^) *Campylobacter* cells, accounting for putative DNA loss during sample isolation.

The comparison of dead bacteria in the presence and absence of PMA led to estimations of whether the reduction of dead cell signal was sufficient for proper quantification of viable bacteria. As expected from the results above, using 20 µM PMA did not lead to sufficient differentiation of viable cells among 99% of dead cells, since the dead cell reduction (ΔCt  = 4.2 or ΔCt  = 2.1 for the two different chicken rinses) did not exceed the measured reduction of the C_t_ value of the sample. However, 50 and 100 µM were suitable to quantify spiked viable bacterial counts as low as 10^2^–10^3^ among log 2 or log 2.5 (for 10^3^) excess of dead bacteria in the tested matrices with maximal deviation of 0.3 log (Tab. 5). As mentioned above (maximally one out of four bacteria formed a CFU, Tab. 1), 10^2^ bacterial counts (here IPIU) per ml of chicken rinse corresponded to maximally 25 CFU/ml under our conditions. Thus, the detection limit was theoretically 25 CFU per g chicken meat or 12.5 CFU per g skin in our experimental setup, given a real-time PCR detection limit of 10 chromosomal DNA copies.

## Discussion

The ISO 10272-2 exclusively detects the *in vitro* culturable state of *Campylobacter*. Previous data revealed an apparent huge discrepancy between the detection of viable bacteria by direct plating compared to enrichment, with respect to prevalence estimation in fresh chicken at retail. During German *Campylobacter* monitoring, approximately 90% of *Campylobacter*-positive fresh chicken at retail were only detected after enrichment, although the theoretical detection limit of the quantitative microbiological detection method was as low as 10 CFU/g [2]. This corroborates the unsuitability of the quantitative parameter “CFU” for assessing the risk for infection by *Campylobacter*.

A dead bacterium is demolished in essential processes of life, e.g. DNA replication, DNA transcription, protein biosynthesis and/or membrane integrity. The most probable cause of death, which the microaerophilic *Campylobacter* encounters outside its ecological niche, is oxidative stress and insufficient protective adaptation under additional cold stress. We showed that oxidative stress exerted by addition of H_2_O_2_ led to significant loss of membrane integrity in *Campylobacter*, detectable by intercalation of dyes in cytoplasmically located DNA. Four states of bacteria were defined using fluorescent tools and flow cytometry: (i) a culturable bacterium, (ii) a metabolically active form, which does not form a CFU, (iii) an inactive but intact bacterium and, (iv) a dye-permeable form with a compromised membrane [16]. All states except that with a compromised membrane may putatively be infectious. In this study, we confirmed that even under normal growth conditions, the CFU of *Campylobacter* varied considerably (>log 2, Tab. 1) for exponential versus stationary phase bacteria, while the bacterial counts and DNA content were fairly stable and PI was mainly excluded from these bacteria. Thus, especially for a fastidious bacterium like *Campylobacter*, the parameter of CFU is deceptive for quantification of the risk of infection. Hence, we suggest a novel definition, namely an “intact and potentially infectious unit” (IPIU), corresponding to *Campylobacter* with an intact membrane, non-permeable by the DNA intercalating dye PI (and PMA). The term IPIU clearly differs from the term “viable but non-culturable bacteria” (VBNC), with the latter merely constituting a sub-population of IPIU.

We unambiguously showed that EtBr and its analogue EMA were not sufficiently excluded from viable cells. In particular, metabolically less active cells from stationary phase were not able to actively exclude the dyes, leading to a significant loss of DNA signal from viable bacteria in the presence of EMA. In addition, entry of EMA into viable *Campylobacter* significantly depended on time and temperature. Simultaneous handling of multiple samples is therefore, hampered by variable signal loss of DNA from viable cells, rendering the EMA real-time PCR method insufficiently robust for routine diagnosis. Moreover, EMA treatment did not account for different metabolic states of the bacteria. Thus, a calibration curve, using highly metabolically active cultures, able to extrude EMA, will undoubtedly lead to underestimation of the load of viable bacteria in native samples with *Campylobacter* of unknown metabolic state. Note that cultivation of *Campylobacter* for 24–48 h in different laboratories does not necessarily lead to the same metabolic state of the majority of the bacteria. Under our conditions, some of the bacteria on 24 h plates did probably already enter stationary growth phase due to quite optimal starting cultures and growth conditions.

In contrast, PMA was excluded from viable *Campylobacter*, irrespective of the metabolic state of the cells and proved suitable as viable/dead discriminatory dye. To our knowledge, these results present the first evidence that PMA- (and not EMA-) exclusion from viable cells is a passive process, explaining the much higher discriminatory power observed for the PMA-PCR method conducted in various bacteria [17]–[20]. As observed by others for *Legionella* [21] and in contrast to previous results with *Campylobacter* [7] PMA was less efficient in inhibiting the DNA signal from dead cells than EMA (Fig. 2). Using higher concentrations of PMA and higher incubation temperatures, we observed that the reduction of the signal from dead cells was increased to levels observed for EMA. Increasing temperatures have also been shown to enhance PMA entry into dead *Listeria* and *Salmonella*, although temperatures of 40°C resulted in entry of PMA into live *Listeria* and temperatures around 30°C were not yet tested [22]. Additionally, our results suggest that the composition of the matrix severely affected dead cell signal reduction. Also lower concentrations of PMA might be sufficient for some matrices, but the degree of dead cell signal reduction should be monitored by implementation of “sample process controls” (SPC) in order to avoid overestimation of viable bacteria. Such an SPC is also essential to calibrate to an absolute, bacterial cell number and account for DNA losses during extraction [23] (see below).

However, our approach of using *C. jejuni* dead cells as “sample process control” still has some limitations and needs optimization for routine application in laboratories. First, four instead of two samples have to be processed per quantification. This does not only entail increased labour and costs but also has the disadvantage of lack of a direct “internal sample process control” (ISPC) within the respective aliquot. Such an ISPC would compensate for heterogeneity of samples, putatively leading to some variations in PMA performance and in loss of DNA during extraction, which cannot be absolutely excluded. Second, the *C. jejuni* dead cell standard has to be freshly generated by the laboratory and might vary in permeability properties and total amount of DNA, which has to be calibrated. Third, quantitative standardization of a high amount of *C. jejuni* cells (10^7^), which is necessary to overcome the native load of *Campylobacter* contamination, is sensitive towards small quantitative deviations in real-time PCR linearity between C_t_ values and target amount.

Further research is necessary in order to define and produce an improved ISPC for the direct monitoring of variations during the processing within the samples quantified by the PMA-based real-time PCR method. Moreover, this reproducible ISPC should exhibit defined and stable membrane permeability properties.

In conclusion, the implementation of IPIU detection instead of or complementary to CFU for monitoring *Campylobacter* food safety will considerably enhance the detection capability along the whole food chain, even when *Campylobacter* are stressed and lose their ability to quantitatively grow on agar plates. Moreover, detection of IPIU might also lead to the revelation of unknown transmission routes of the pathogen. With respect to the implementation of a proposed “microbiological criterion” of 500 to 1000 CFU/g skin of poultry carcasses at slaughterhouse, data have to be reevaluated in the context of the IPIU. The recently published quantitative data on *Campylobacter* from slaughterhouse samples using PMA [8] should be extended, using efficient dead cell signal reduction with SPC (or prospectively ISPC) controls.

## References

[1] El-Shibiny A, Connerton P, Connerton I (2009) Survival at refrigeration and freezing temperatures of *Campylobacter coli* and *Campylobacter jejuni* on chicken skin applied as axenic and mixed inoculums. Int J Food Microbiol 131: 197–202. 19324444 10.1016/j.ijfoodmicro.2009.02.024

[2] Stingl K, Knüver M-T, Vogt P, Buhler C, Krüger N-J, et al (2012) Quo vadis? - Monitoring *Campylobacter* in Germany. Eur J Microbiol Immunol 2: 88–96. 10.1556/EuJMI.2.2012.1.12 PMC3933994 24611125

[3] EFSA (2011) Scientific Opinion on *Campylobacter* in broiler meat production: control options and performance objectives and/or targets at different stages of the food chain. EFSA Journal 9: 2105.

[4] Bolton PH, Kearns DR (1978) Spectroscopic properties of ethidium monoazide: a fluorescent photoaffinity label for nucleic acids. Nucleic Acids Res 5: 4891–4903. 745997 10.1093/nar/5.12.4891 PMC342796

[5] Nogva HK, Dromtorp SM, Nissen H, Rudi K (2003) Ethidium monoazide for DNA-based differentiation of viable and dead bacteria by 5′-nuclease PCR. Biotechniques 34: 804–3. 12703305 10.2144/03344rr02

[6] Rudi K, Moen B, Dromtorp SM, Holck AL (2005) Use of ethidium monoazide and PCR in combination for quantification of viable and dead cells in complex samples. Appl Environ Microbiol 71: 1018–1024. 15691961 10.1128/AEM.71.2.1018-1024.2005 PMC546808

[7] Josefsen MH, Lofstrom C, Hansen TB, Christensen LS, Olsen JE, et al (2010) Rapid quantification of viable *Campylobacter* bacteria on chicken carcasses, using real-time PCR and propidium monoazide treatment, as a tool for quantitative risk assessment. Appl Environ Microbiol 76: 5097–5104. 20562292 10.1128/AEM.00411-10 PMC2916463

[8] Pacholewicz E, Swart A, Lipman LJ, Wagenaar JA, Havelaar AH, et al (2013) Propidium monoazide does not fully inhibit the detection of dead *Campylobacter* on broiler chicken carcasses by qPCR. J Microbiol Methods 95: 32–38. 23811205 10.1016/j.mimet.2013.06.003

[9] Fittipaldi M, Nocker A, Codony F (2012) Progress in understanding preferential detection of live cells using viability dyes in combination with DNA amplification. J Microbiol Methods 91: 276–289. 22940102 10.1016/j.mimet.2012.08.007

[10] Rossmanith P, Roder B, Fruhwirth K, Vogl C, Wagner M (2011) Mechanisms of degradation of DNA standards for calibration function during storage. Appl Microbiol Biotechnol 89: 407–417. 20967442 10.1007/s00253-010-2943-2

[11] Lübeck PS, Wolffs P, On SL, Ahrens P, Radstrom P, et al (2003) Toward an international standard for PCR-based detection of food-borne thermotolerant Campylobacters: assay development and analytical validation. Appl Environ Microbiol 69: 5664–5669. 12957958 10.1128/AEM.69.9.5664-5669.2003 PMC194918

[12] Josefsen MH, Jacobsen NR, Hoorfar J (2004) Enrichment followed by quantitative PCR both for rapid detection and as a tool for quantitative risk assessment of food-borne thermotolerant campylobacters. Appl Environ Microbiol 70: 3588–3592. 15184161 10.1128/AEM.70.6.3588-3592.2004 PMC427791

[13] Anderson A, Pietsch K, Zucker R, Mayr A, Müller-Hohe E, et al (2011) Validation of a duplex real-time PCR for the detection of *Salmonella* spp. in different food products. Food Anal Methods 4: 259–267.

[14] van Alphen LB, Burt SA, Veenendaal AK, Bleumink-Pluym NM, van Putten JP (2012) The natural antimicrobial carvacrol inhibits Campylobacter jejuni motility and infection of epithelial cells. PLoS One 7: e45343. 23049787 10.1371/journal.pone.0045343 PMC3458047

[15] Mamelli L, Prouzet-Mauleon V, Pages JM, Megraud F, Bolla JM (2005) Molecular basis of macrolide resistance in Campylobacter: role of efflux pumps and target mutations. J Antimicrob Chemother 56: 491–497. 16055509 10.1093/jac/dki253

[16] Nebe-von-Caron G, Stephens PJ, Hewitt CJ, Powell JR, Badley RA (2000) Analysis of bacterial function by multi-colour fluorescence flow cytometry and single cell sorting. J Microbiol Methods 42: 97–114. 11000436 10.1016/s0167-7012(00)00181-0

[17] Cawthorn DM, Witthuhn RC (2008) Selective PCR detection of viable *Enterobacter sakazakii* cells utilizing propidium monoazide or ethidium bromide monoazide. J Appl Microbiol 105: 1178–1185. 18624747 10.1111/j.1365-2672.2008.03851.x

[18] Flekna G, Stefanic P, Wagner M, Smulders FJ, Mozina SS, et al (2007) Insufficient differentiation of live and dead *Campylobacter jejuni* and *Listeria monocytogenes* cells by ethidium monoazide (EMA) compromises EMA/real-time PCR. Res Microbiol 158: 405–412. 17449228 10.1016/j.resmic.2007.02.008

[19] Kobayashi H, Oethinger M, Tuohy MJ, Hall GS, Bauer TW (2009) Unsuitable distinction between viable and dead *Staphylococcus aureus* and *Staphylococcus epidermidis* by ethidium bromide monoazide. Lett Appl Microbiol 48: 633–638. 19416465 10.1111/j.1472-765X.2009.02585.x

[20] Loozen G, Boon N, Pauwels M, Quirynen M, Teughels W (2011) Live/dead real-time polymerase chain reaction to assess new therapies against dental plaque-related pathologies. Mol Oral Microbiol 26: 253–261. 21729246 10.1111/j.2041-1014.2011.00615.x

[21] Chang B, Taguri T, Sugiyama K, Amemura-Maekawa J, Kura F, et al (2010) Comparison of ethidium monoazide and propidium monoazide for the selective detection of viable Legionella cells. Jpn J Infect Dis 63: 119–123. 20332575

[22] Nkuipou-Kenfack E, Engel H, Fakih S, Nocker A (2013) Improving efficiency of viability-PCR for selective detection of live cells. J Microbiol Methods 93: 20–24. 23389080 10.1016/j.mimet.2013.01.018

[23] Rossmanith P, Mester P, Fruhwirth K, Fuchs S, Wagner M (2011) Proof of concept for recombinant cellular controls in quantitative molecular pathogen detection. Appl Environ Microbiol 77: 2531–2533. 21317266 10.1128/AEM.02601-10 PMC3067449

